# Supplementary material for: Interpretable multivariate survival models: Improving predictions for conversion from mild cognitive impairment to Alzheimer’s disease via data fusion and machine learning
Source: PLoS One. 2026 Apr 30;21(4):e0321671. doi: 10.1371/journal.pone.0321671 (PMC13132236; doi:10.1371/journal.pone.0321671)
Supplement: S1 File — Additional information. Experimental data, definitions of abbreviations, and calibration plots are provided in the Supplementary Material. (PDF) [file pone.0321671.s001.pdf]

Supplementary Material

For the Paper: Interpretable multivariate survival models: Improving predictions for conversion from mild cognitive impairment to Alzheimer's disease via data fusion and machine learning: Supplementary materials

Authors: Diana Sofia Rosales-Gurmendi[1], Gerardo Alejandro Fumagal-González[1*] Jorge Orozco[1], Joshua Farber[2], Victor Treviño[3], Emmanuel Martinez-Ledesma[3,4], Antonio Martinez-Torteya[5], Fabiola Rosales-Gurmendi[6], Jose Tamez-Peña.[3] and for the Alzheimer's Disease Neuroimaging Initiative.[**]

1 School of Engineering and Sciences, Tecnologico de Monterrey, Monterrey, Mexico.
2 Qmetrics Technologies LLC, Rochester, NY, USA.
3 School of Medicine and Health Sciences, Tecnologico de Monterrey, Monterrey, Mexico.
4 The Institute for Obesity Research, Tecnologico de Monterrey, Monterrey, Mexico.
5 Engineering Department, Universidad de Monterrey, Monterrey, Mexico.
6 School of Medicine, Universidad Peruana Cayetano Heredia, Lima, Peru.

* gerardofumagalgzz@gmail.com
**Membership of the Alzheimer's Disease Neuroimaging Initiative is listed in the Acknowledgments

Supplementary Material

This document provides additional details, tables, and figures to supplement the main findings of the paper. The content is organized to support the analysis and results presented in the main manuscript.

0.1 Github Repository

Available Github repository can be found here: https://github.com/joseTamezPena/SurvivalTadpole Readers are encouraged to consult this resource for a comprehensive understanding of the study methodology and reproducibility of the results.

0.2 Abbreviations and Definitions Related

Table 1: Abbreviations and Definitions Related to Alzheimer's Disease Research

**Feature**	**Description**
Aβ	amyloid beta
ACC	accuracy
AD	Alzheimer's disease
ADAS	Alzheimer's Disease Assessment Scale
ADNI	Alzheimer's Disease Neuroimaging Initiative
APOE ε4	apolipoprotein ε4
AUC	area under the curve
BESS	Best subset selection
BIC	Bayesian information criterion
BSWiMS	Bootstrap Stepwise Model Selection
CDRSB	Clinical Dementia Rating Sum of Boxes
CI	confidence intervals
CSF	cerebrospinal fluid
FAQ	Functional Assessment Questionnaire
FDR	false discovery rate
FS	future selection
GPDAS	golden section primal-dual active set
HR	hazard ratios
ICV	Intracranial volume
KM	Kaplan-Meier
LASSO	Least Absolute Shrinkage and Selection Operator
LIP	Left Inferior Parietal
MCI	mild cognitive impairment
ML	machine learning
MMSE	Mini-Mental State Examination
MRI	magneto resonance
PET	Positron-emitting tomography
p-tau	phosphorylated tau
RAVLT	Rey's Auditory Verbal Learning Test
RHOCV	repeated holdout cross-validation

Table 1: Abbreviations and Definitions Related to Alzheimer's Disease Research

Feature	Description
ROC	receiver operating characteristic
SEN	sensitivity
SL	Statistical learning
SPDAS	sequential primal-dual active set
SPE	specificity
TADPOLE	The Alzheimer's Disease Prediction Of Longitudinal Evolution
TN	true negative
TP	true positive
UCSF	University of California San Francisco

0.3 List of Selected TADPOLE MCI Features

Table 2: Names and Definitions of the TADPOLE MCI Features

Feature	Description
AGE	Age
Gender	Gender
ADAS11	Alzheimer's Disease Assessment Scale 11
ADAS13	Alzheimer's Disease Assessment Scale 13
MMSE	Mini-Mental State Examination
RAVLT immediate	Rey's Auditory Verbal Learning Test immediate
RAVLT learning	Rey's Auditory Verbal Learning Test learning
RAVLT forgetting	Rey's Auditory Verbal Learning Test forgetting
RAVLT perc forgetting	Rey's Auditory Verbal Learning Test percentage forgettings
FAQ	Functional Assessment Questionnaire
APOE4	apolipoprotein Epsilon 4
Ventricles	Ventricles
WholeBrain	WholeBrain
ICV	Intracranial volume
ABETA	Amyloid-Beta
TAU	microtubule-associated protein
PTAU	phosphorylated tau
ST10CV	Volume (Cortical Parcellation) of Icv
ST127SV	Volume (WM Parcellation) of ThirdVentricle
ST128SV	Volume (WM Parcellation) of WMHypoIntensities
ST1SV	Volume (WM Parcellation) of Brainstem
ST2SV	Volume (WM Parcellation) of CorpusCallosumAnterior
ST3SV	Volume (WM Parcellation) of CorpusCallosumCentral

Table 2: Names and Definitions of the TADPOLE MCI Features

Feature	Description
ST4SV	Volume (WM Parcellation) of CorpusCallosumMidAnterior
ST5SV	Volume (WM Parcellation) of CorpusCallosumMidPosterior
ST68SV	Volume (WM Parcellation) of NonWMHypoIntensities
ST69SV	Volume (WM Parcellation) of OpticChiasm
ST6SV	Volume (WM Parcellation) of CorpusCallosumPosterior
ST7SV	Volume (WM Parcellation) of Csf
ST9SV	Volume (WM Parcellation) of FourthVentricle
MST13TA	Cortical Thickness Average of LeftBankssts
MST14TA	Cortical Thickness Average of LeftCaudalAnteriorCingulate
MST15TA	Cortical Thickness Average of LeftCaudalMiddleFrontal
MST23TA	Cortical Thickness Average of LeftCuneus
MST24TA	Cortical Thickness Average of LeftEntorhinal
MST25TA	Cortical Thickness Average of LeftFrontalPole
MST26TA	Cortical Thickness Average of LeftFusiform
MST31TA	Cortical Thickness Average of LeftInferiorParietal
MST32TA	Cortical Thickness Average of LeftInferiorTemporal
MST129TA	Cortical Thickness Average of LeftInsula
MST34TA	Cortical Thickness Average of LeftIsthmusCingulate
MST35TA	Cortical Thickness Average of LeftLateralOccipital
MST36TA	Cortical Thickness Average of LeftLateralOrbitofrontal
MST38TA	Cortical Thickness Average of LeftLingual
MST39TA	Cortical Thickness Average of LeftMedialOrbitofrontal
MST40TA	Cortical Thickness Average of LeftMiddleTemporal
MST43TA	Cortical Thickness Average of LeftParacentral
MST44TA	Cortical Thickness Average of LeftParahippocampal
MST45TA	Cortical Thickness Average of LeftParsOpercularis
MST46TA	Cortical Thickness Average of LeftParsOrbitalis
MST47TA	Cortical Thickness Average of LeftParsTriangularis
MST48TA	Cortical Thickness Average of LeftPericalcarine
MST49TA	Cortical Thickness Average of LeftPostcentral
MST50TA	Cortical Thickness Average of LeftPosteriorCingulate
MST51TA	Cortical Thickness Average of LeftPrecentral
MST52TA	Cortical Thickness Average of LeftPrecuneus
MST54TA	Cortical Thickness Average of LeftRostralAnteriorCingulate
MST55TA	Cortical Thickness Average of LeftRostralMiddleFrontal
MST56TA	Cortical Thickness Average of LeftSuperiorFrontal
MST57TA	Cortical Thickness Average of LeftSuperiorParietal
MST58TA	Cortical Thickness Average of LeftSuperiorTemporal
MST59TA	Cortical Thickness Average of LeftSupramarginal
MST60TA	Cortical Thickness Average of LeftTemporalPole
MST62TA	Cortical Thickness Average of LeftTransverseTemporal

Table 2: Names and Definitions of the TADPOLE MCI Features

Feature	Description
MST13TS	Cortical Thickness Standard Deviation of LeftBankssts
MST14TS	Cortical Thickness Standard Deviation of LeftCaudalAnteriorCingulate
MST15TS	Cortical Thickness Standard Deviation of LeftCaudalMiddleFrontal
MST23TS	Cortical Thickness Standard Deviation of LeftCuneus
MST24TS	Cortical Thickness Standard Deviation of LeftEntorhinal
MST25TS	Cortical Thickness Standard Deviation of LeftFrontalPole
MST26TS	Cortical Thickness Standard Deviation of LeftFusiform
MST31TS	Cortical Thickness Standard Deviation of LeftInferiorParietal
MST32TS	Cortical Thickness Standard Deviation of LeftInferiorTemporal
MST129TS	Cortical Thickness Standard Deviation of LeftInsula
MST34TS	Cortical Thickness Standard Deviation of LeftIsthmusCingulate
MST35TS	Cortical Thickness Standard Deviation of LeftLateralOccipital
MST36TS	Cortical Thickness Standard Deviation of LeftLateralOrbitofrontal
MST38TS	Cortical Thickness Standard Deviation of LeftLingual
MST39TS	Cortical Thickness Standard Deviation of LeftMedialOrbitofrontal
MST40TS	Cortical Thickness Standard Deviation of LeftMiddleTemporal
MST43TS	Cortical Thickness Standard Deviation of LeftParacentral
MST44TS	Cortical Thickness Standard Deviation of LeftParahippocampal
MST45TS	Cortical Thickness Standard Deviation of LeftParsOpercularis
MST46TS	Cortical Thickness Standard Deviation of LeftParsOrbitalis
MST47TS	Cortical Thickness Standard Deviation of LeftParsTriangularis
MST48TS	Cortical Thickness Standard Deviation of LeftPericalcarine
MST49TS	Cortical Thickness Standard Deviation of LeftPostcentral
MST50TS	Cortical Thickness Standard Deviation of LeftPosteriorCingulate
MST51TS	Cortical Thickness Standard Deviation of LeftPrecentral
MST52TS	Cortical Thickness Standard Deviation of LeftPrecuneus

Table 2: Names and Definitions of the TADPOLE MCI Features

Feature	Description
MST54TS	Cortical Thickness Standard Deviation of LeftRostralAnteriorCingulate
MST55TS	Cortical Thickness Standard Deviation of LeftRostralMiddleFrontal
MST56TS	Cortical Thickness Standard Deviation of LeftSuperiorFrontal
MST57TS	Cortical Thickness Standard Deviation of LeftSuperiorParietal
MST58TS	Cortical Thickness Standard Deviation of LeftSuperiorTemporal
MST59TS	Cortical Thickness Standard Deviation of LeftSupramarginal
MST60TS	Cortical Thickness Standard Deviation of LeftTemporalPole
MST62TS	Cortical Thickness Standard Deviation of LeftTransverseTemporal
MST13SA	Surface Area of LeftBankssts
MST14SA	Surface Area of LeftCaudalAnteriorCingulate
MST15SA	Surface Area of LeftCaudalMiddleFrontal
MST23SA	Surface Area of LeftCuneus
MST24SA	Surface Area of LeftEntorhinal
MST25SA	Surface Area of LeftFrontalPole
MST26SA	Surface Area of LeftFusiform
MST31SA	Surface Area of LeftInferiorParietal
MST32SA	Surface Area of LeftInferiorTemporal
MST129SA	Surface Area of LeftInsula
MST34SA	Surface Area of LeftIsthmusCingulate
MST35SA	Surface Area of LeftLateralOccipital
MST36SA	Surface Area of LeftLateralOrbitofrontal
MST38SA	Surface Area of LeftLingual
MST39SA	Surface Area of LeftMedialOrbitofrontal
MST40SA	Surface Area of LeftMiddleTemporal
MST43SA	Surface Area of LeftParacentral
MST44SA	Surface Area of LeftParahippocampal
MST45SA	Surface Area of LeftParsOpercularis
MST46SA	Surface Area of LeftParsOrbitalis
MST47SA	Surface Area of LeftParsTriangularis
MST48SA	Surface Area of LeftPericalcarine
MST49SA	Surface Area of LeftPostcentral
MST50SA	Surface Area of LeftPosteriorCingulate
MST51SA	Surface Area of LeftPrecentral
MST52SA	Surface Area of LeftPrecuneus
MST54SA	Surface Area of LeftRostralAnteriorCingulate
MST55SA	Surface Area of LeftRostralMiddleFrontal
MST56SA	Surface Area of LeftSuperiorFrontal

Table 2: Names and Definitions of the TADPOLE MCI Features

Feature	Description
MST57SA	Surface Area of LeftSuperiorParietal
MST58SA	Surface Area of LeftSuperiorTemporal
MST59SA	Surface Area of LeftSupramarginal
MST60SA	Surface Area of LeftTemporalPole
MST62SA	Surface Area of LeftTransverseTemporal
MST13CV	Volume (Cortical Parcellation) of LeftBankssts
MST14CV	Volume (Cortical Parcellation) of LeftCaudalAnteriorCingulate
MST15CV	Volume (Cortical Parcellation) of LeftCaudalMiddleFrontal
MST23CV	Volume (Cortical Parcellation) of LeftCuneus
MST24CV	Volume (Cortical Parcellation) of LeftEntorhinal
MST25CV	Volume (Cortical Parcellation) of LeftFrontalPole
MST26CV	Volume (Cortical Parcellation) of LeftFusiform
MST31CV	Volume (Cortical Parcellation) of LeftHemisphereWM
MST32CV	Volume (Cortical Parcellation) of LeftInferiorTemporal
MST129CV	Volume (Cortical Parcellation) of LeftInsula
MST34CV	Volume (Cortical Parcellation) of LeftIsthmusCingulate
MST35CV	Volume (Cortical Parcellation) of LeftLateralOccipital
MST36CV	Volume (Cortical Parcellation) of LeftLateralOrbitofrontal
MST38CV	Volume (Cortical Parcellation) of LeftLingual
MST39CV	Volume (Cortical Parcellation) of LeftMedialOrbitofrontal
MST40CV	Volume (Cortical Parcellation) of LeftMiddleTemporal
MST43CV	Volume (Cortical Parcellation) of LeftParacentral
MST44CV	Volume (Cortical Parcellation) of LeftParahippocampal
MST45CV	Volume (Cortical Parcellation) of LeftParsOpercularis
MST46CV	Volume (Cortical Parcellation) of LeftParsOrbitalis
MST47CV	Volume (Cortical Parcellation) of LeftParsTriangularis
MST48CV	Volume (Cortical Parcellation) of LeftPericalcarine
MST49CV	Volume (Cortical Parcellation) of LeftPostcentral
MST50CV	Volume (Cortical Parcellation) of LeftPosteriorCingulate
MST51CV	Volume (Cortical Parcellation) of LeftPrecentral
MST52CV	Volume (Cortical Parcellation) of LeftPrecuneus
MST54CV	Volume (Cortical Parcellation) of LeftRostralAnteriorCingulate
MST55CV	Volume (Cortical Parcellation) of LeftRostralMiddleFrontal
MST56CV	Volume (Cortical Parcellation) of LeftSuperiorFrontal
MST57CV	Volume (Cortical Parcellation) of LeftSuperiorParietal
MST58CV	Volume (Cortical Parcellation) of LeftSuperiorTemporal
MST59CV	Volume (Cortical Parcellation) of LeftSupramarginal
MST60CV	Volume (Cortical Parcellation) of LeftTemporalPole

Table 2: Names and Definitions of the TADPOLE MCI Features

Feature	Description
MST62CV	Volume (Cortical Parcellation) of LeftTransverseTemporal
MST11SV	Volume (WM Parcellation) of LeftAccumbensArea
MST12SV	Volume (WM Parcellation) of LeftAmygdala
MST16SV	Volume (WM Parcellation) of LeftCaudate
MST17SV	Volume (WM Parcellation) of LeftCerebellumCortex
MST18SV	Volume (WM Parcellation) of LeftCerebellumWM
MST21SV	Volume (WM Parcellation) of LeftChoroidPlexus
MST29SV	Volume (WM Parcellation) of LeftHippocampus
MST30SV	Volume (WM Parcellation) of LeftInferiorLateralVentricle
MST37SV	Volume (WM Parcellation) of LeftLateralVentricle
MST42SV	Volume (WM Parcellation) of LeftPallidum
MST53SV	Volume (WM Parcellation) of LeftPutamen
MST61SV	Volume (WM Parcellation) of LeftThalamus
MST65SV	Volume (WM Parcellation) of LeftVentralDC
MST66SV	Volume (WM Parcellation) of LeftVessel
RDST13TA	Cortical Thickness Average of LeftBankssts
RDST14TA	Cortical Thickness Average of LeftCaudalAnteriorCingulate
RDST15TA	Cortical Thickness Average of LeftCaudalMiddleFrontal
RDST23TA	Cortical Thickness Average of LeftCuneus
RDST24TA	Cortical Thickness Average of LeftEntorhinal
RDST25TA	Cortical Thickness Average of LeftFrontalPole
RDST26TA	Cortical Thickness Average of LeftFusiform
RDST31TA	Cortical Thickness Average of LeftInferiorParietal
RDST32TA	Cortical Thickness Average of LeftInferiorTemporal
RDST129TA	Cortical Thickness Average of LeftInsula
RDST34TA	Cortical Thickness Average of LeftIsthmusCingulate
RDST35TA	Cortical Thickness Average of LeftLateralOccipital
RDST36TA	Cortical Thickness Average of LeftLateralOrbitofrontal
RDST38TA	Cortical Thickness Average of LeftLingual
RDST39TA	Cortical Thickness Average of LeftMedialOrbitofrontal
RDST40TA	Cortical Thickness Average of LeftMiddleTemporal
RDST43TA	Cortical Thickness Average of LeftParacentral
RDST44TA	Cortical Thickness Average of LeftParahippocampal
RDST45TA	Cortical Thickness Average of LeftParsOpercularis
RDST46TA	Cortical Thickness Average of LeftParsOrbitalis
RDST47TA	Cortical Thickness Average of LeftParsTriangularis
RDST48TA	Cortical Thickness Average of LeftPericalcarine
RDST49TA	Cortical Thickness Average of LeftPostcentral
RDST50TA	Cortical Thickness Average of LeftPosteriorCingulate
RDST51TA	Cortical Thickness Average of LeftPrecentral
RDST52TA	Cortical Thickness Average of LeftPrecuneus

Table 2: Names and Definitions of the TADPOLE MCI Features

Feature	Description
RDST54TA	Cortical Thickness Average of LeftRostralAnteriorCingulate
RDST55TA	Cortical Thickness Average of LeftRostralMiddleFrontal
RDST56TA	Cortical Thickness Average of LeftSuperiorFrontal
RDST57TA	Cortical Thickness Average of LeftSuperiorParietal
RDST58TA	Cortical Thickness Average of LeftSuperiorTemporal
RDST59TA	Cortical Thickness Average of LeftSupramarginal
RDST60TA	Cortical Thickness Average of LeftTemporalPole
RDST62TA	Cortical Thickness Average of LeftTransverseTemporal
RDST13TS	Cortical Thickness Standard Deviation of LeftBankssts
RDST14TS	Cortical Thickness Standard Deviation of LeftCaudalAnteriorCingulate
RDST15TS	Cortical Thickness Standard Deviation of LeftCaudalMiddleFrontal
RDST23TS	Cortical Thickness Standard Deviation of LeftCuneus
RDST24TS	Cortical Thickness Standard Deviation of LeftEntorhinal
RDST25TS	Cortical Thickness Standard Deviation of LeftFrontalPole
RDST26TS	Cortical Thickness Standard Deviation of LeftFusiform
RDST31TS	Cortical Thickness Standard Deviation of LeftInferiorParietal
RDST32TS	Cortical Thickness Standard Deviation of LeftInferiorTemporal
RDST129TS	Cortical Thickness Standard Deviation of LeftInsula
RDST34TS	Cortical Thickness Standard Deviation of LeftIsthmusCingulate
RDST35TS	Cortical Thickness Standard Deviation of LeftLateralOccipital
RDST36TS	Cortical Thickness Standard Deviation of LeftLateralOrbitofrontal
RDST38TS	Cortical Thickness Standard Deviation of LeftLingual
RDST39TS	Cortical Thickness Standard Deviation of LeftMedialOrbitofrontal
RDST40TS	Cortical Thickness Standard Deviation of LeftMiddleTemporal
RDST43TS	Cortical Thickness Standard Deviation of LeftParacentral
RDST44TS	Cortical Thickness Standard Deviation of LeftParahippocampal
RDST45TS	Cortical Thickness Standard Deviation of LeftParsOpercularis
RDST46TS	Cortical Thickness Standard Deviation of LeftParsOrbitalis

Table 2: Names and Definitions of the TADPOLE MCI Features

Feature	Description
RDST47TS	Cortical Thickness Standard Deviation of LeftParsTriangularis
RDST48TS	Cortical Thickness Standard Deviation of LeftParsTriangularis
RDST49TS	Cortical Thickness Standard Deviation of LeftPostcentral
RDST50TS	Cortical Thickness Standard Deviation of LeftPosteriorCingulate
RDST51TS	Cortical Thickness Standard Deviation of LeftPrecentral
RDST52TS	Cortical Thickness Standard Deviation of LeftPrecuneus
RDST54TS	Cortical Thickness Standard Deviation of LeftRostralAnteriorCingulate
RDST55TS	Cortical Thickness Standard Deviation of LeftRostralMiddleFrontal
RDST56TS	Cortical Thickness Standard Deviation of LeftSuperiorFrontal
RDST57TS	Cortical Thickness Standard Deviation of LeftSuperiorParietal
RDST58TS	Cortical Thickness Standard Deviation of LeftSuperiorTemporal
RDST59TS	Cortical Thickness Standard Deviation of LeftSupramarginal
RDST60TS	Cortical Thickness Standard Deviation of LeftTemporalPole
RDST62TS	Cortical Thickness Standard Deviation of LeftTransverseTemporal
RDST13SA	Surface Area of LeftBankssts
RDST14SA	Surface Area of LeftCaudalAnteriorCingulate
RDST15SA	Surface Area of LeftCaudalMiddleFrontal
RDST23SA	Surface Area of LeftCuneus
RDST24SA	Surface Area of LeftEntorhinal
RDST25SA	Surface Area of LeftFrontalPole
RDST26SA	Surface Area of LeftFusiform
RDST31SA	Surface Area of LeftInferiorParietal
RDST32SA	Surface Area of LeftInferiorTemporal
RDST129SA	Surface Area of LeftInsula
RDST34SA	Surface Area of LeftIsthmusCingulate
RDST35SA	Surface Area of LeftLateralOccipital
RDST36SA	Surface Area of LeftLateralOrbitofrontal
RDST38SA	Surface Area of LeftLingual
RDST39SA	Surface Area of LeftMedialOrbitofrontal
RDST40SA	Surface Area of LeftMiddleTemporal
RDST43SA	Surface Area of LeftParacentral
RDST44SA	Surface Area of LeftParahippocampal
RDST45SA	Surface Area of LeftParsOpercularis

Table 2: Names and Definitions of the TADPOLE MCI Features

Feature	Description
RDST46SA	Surface Area of LeftParsOrbitalis
RDST47SA	Surface Area of LeftParsTriangularis
RDST48SA	Surface Area of LeftPericalcarine
RDST49SA	Surface Area of LeftPostcentral
RDST50SA	Surface Area of LeftPosteriorCingulate
RDST51SA	Surface Area of LeftPrecentral
RDST52SA	Surface Area of LeftPrecuneus
RDST54SA	Surface Area of LeftRostralAnteriorCingulate
RDST55SA	Surface Area of LeftRostralMiddleFrontal
RDST56SA	Surface Area of LeftSuperiorFrontal
RDST57SA	Surface Area of LeftSuperiorParietal
RDST58SA	Surface Area of LeftSuperiorTemporal
RDST59SA	Surface Area of LeftSupramarginal
RDST60SA	Surface Area of LeftTemporalPole
RDST62SA	Surface Area of LeftTransverseTemporal
RDST13CV	Volume (Cortical Parcellation) of LeftBankssts
RDST14CV	Volume (Cortical Parcellation) of LeftCaudalAnteriorCingulate
RDST15CV	Volume (Cortical Parcellation) of LeftCaudalMiddleFrontal
RDST23CV	Volume (Cortical Parcellation) of LeftCuneus
RDST24CV	Volume (Cortical Parcellation) of LeftEntorhinal
RDST25CV	Volume (Cortical Parcellation) of LeftFrontalPole
RDST26CV	Volume (Cortical Parcellation) of LeftFusiform
RDST31CV	Volume (Cortical Parcellation) of LeftInferiorParietal
RDST32CV	Volume (Cortical Parcellation) of LeftInferiorTemporal
RDST129CV	Volume (Cortical Parcellation) of LeftInsula
RDST34CV	Volume (Cortical Parcellation) of LeftIsthmusCingulate
RDST35CV	Volume (Cortical Parcellation) of LeftLateralOccipital
RDST36CV	Volume (Cortical Parcellation) of LeftLateralOrbitofrontal
RDST38CV	Volume (Cortical Parcellation) of LeftLingual
RDST39CV	Volume (Cortical Parcellation) of LeftMedialOrbitofrontal
RDST40CV	Volume (Cortical Parcellation) of LeftMiddleTemporal
RDST43CV	Volume (Cortical Parcellation) of LeftParacentral
RDST44CV	Volume (Cortical Parcellation) of LeftParahippocampal
RDST45CV	Volume (Cortical Parcellation) of LeftParsOpercularis
RDST46CV	Volume (Cortical Parcellation) of LeftParsOrbitalis
RDST47CV	Volume (Cortical Parcellation) of LeftParsTriangularis
RDST48CV	Volume (Cortical Parcellation) of LeftPericalcarine
RDST49CV	Volume (Cortical Parcellation) of LeftPostcentral
RDST50CV	Volume (Cortical Parcellation) of LeftPosteriorCingulate
RDST51CV	Volume (Cortical Parcellation) of LeftPrecentral

Table 2: Names and Definitions of the TADPOLE MCI Features

Feature	Description
RDST52CV	Volume (Cortical Parcellation) of LeftPrecuneus
RDST54CV	Volume (Cortical Parcellation) of LeftRostralAnteriorCingulate
RDST55CV	Volume (Cortical Parcellation) of LeftRostralMiddleFrontal
RDST56CV	Volume (Cortical Parcellation) of LeftSuperiorFrontal
RDST57CV	Volume (Cortical Parcellation) of LeftSuperiorParietal
RDST58CV	Volume (Cortical Parcellation) of LeftSuperiorTemporal
RDST59CV	Volume (Cortical Parcellation) of LeftSupramarginal
RDST60CV	Volume (Cortical Parcellation) of LeftTemporalPole
RDST62CV	Volume (Cortical Parcellation) of LeftTransverseTemporal
RDST11SV	Volume (WM Parcellation) of LeftAccumbensArea
RDST12SV	Volume (WM Parcellation) of LeftAmygdala
RDST16SV	Volume (WM Parcellation) of LeftCaudate
RDST17SV	Volume (WM Parcellation) of LeftCerebellumCortex
RDST18SV	Volume (WM Parcellation) of LeftCerebellumCortex
RDST21SV	Volume (WM Parcellation) of LeftChoroidPlexus
RDST29SV	Volume (WM Parcellation) of LeftHippocampus
RDST30SV	Volume (WM Parcellation) of LeftInferiorLateralVentricle
RDST37SV	Volume (WM Parcellation) of LeftLateralVentricle
RDST42SV	Volume (WM Parcellation) of LeftPallidum
RDST53SV	Volume (WM Parcellation) of LeftPutamen
RDST61SV	Volume (WM Parcellation) of LeftThalamus
RDST65SV	Volume (WM Parcellation) of LeftVentralDC
RDST66SV	Volume (WM Parcellation) of LeftVessel

0.4 Additional Figures

The following figure illustrates the calibration plots for the LASSO, BSWIMS, BESS BIS models.

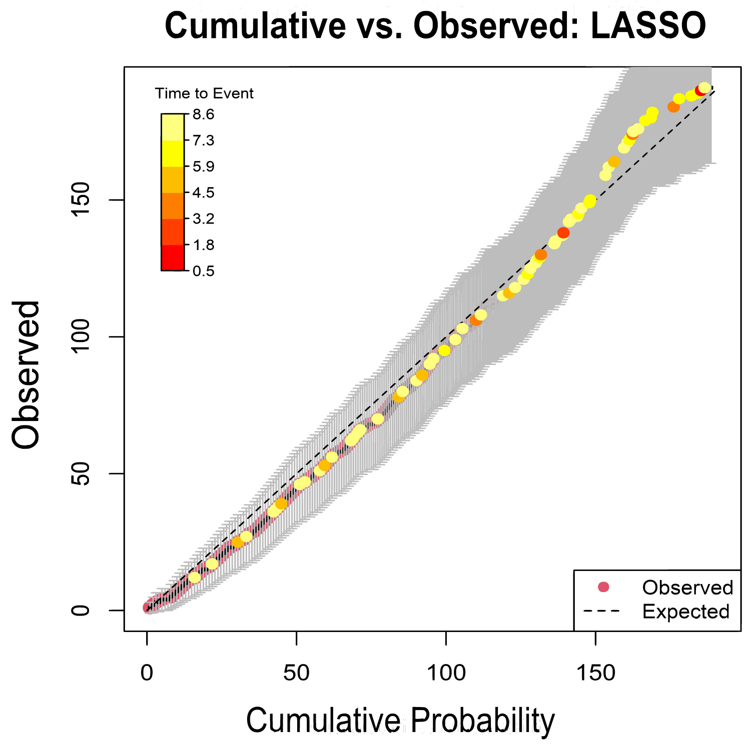

Figure 1: Calibration plot of LASSO Cox model

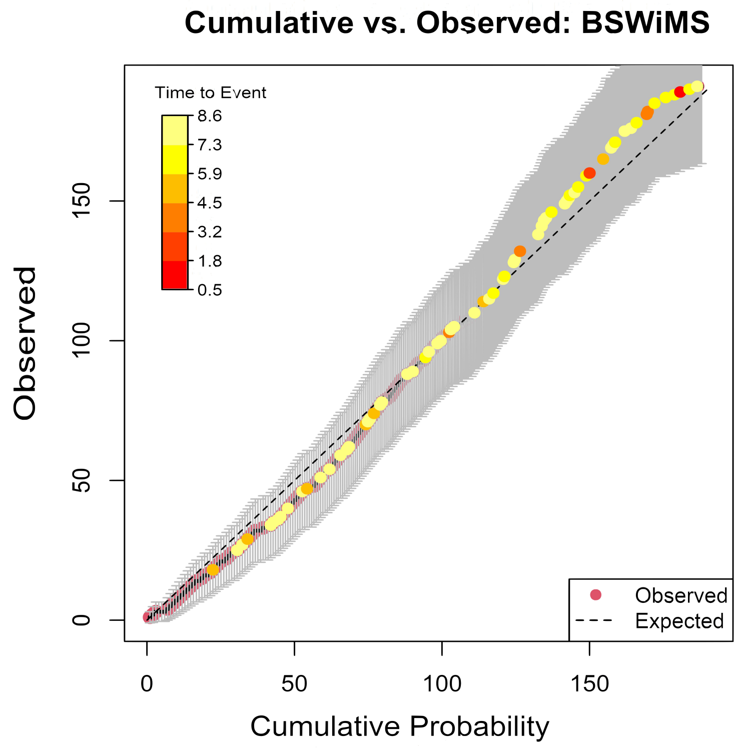

Figure 2: Calibration plot of BSWIMS Cox model

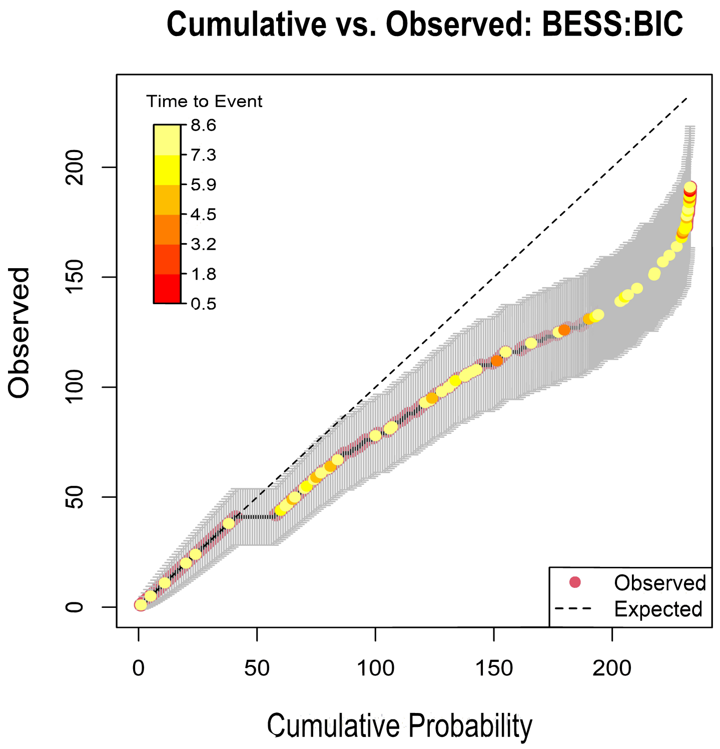

Figure 3: Calibration plot of BESS BIC Cox model
